# MDVs bridge metabolite signals to mitochondrial fitness

**DOI:** 10.1093/lifemeta/loaf022

**Published:** 2025-06-07

**Authors:** Guang Lu, Han-Ming Shen

**Affiliations:** Zhongshan School of Medicine, Sun Yat-sen University, Guangzhou, Guangdong 510080, China; Faculty of Healthy Sciences, Ministry of Education Frontiers Science Center for Precision Oncology, University of Macau, Macao 999078, China

**Emerging evidence suggests that metabolic signals regulate mitochondrial homeostasis, with mitochondria-derived vesicles (MDVs) serving as a critical link between metabolites and mitochondrial quality control. In a recent study, Tang** ***et al*** **. uncovered a novel mechanism in which metabolites modulate mitochondrial homeostasis through** **β** **-hydroxybutyrylation of sorting nexin 9 (SNX9), thereby promoting MDV biogenesis [1].**

Mitochondria stand as versatile organelles intricately engaged in a multitude of life-essential processes, including metabolism, bioenergetics, and cell fate decisions. Therefore, dysfunctions of mitochondria are closely associated with various human diseases such as neurodegenerative diseases. On one hand, to maintain mitochondrial homeostasis, cells have evolved various highly regulated mechanisms for mitochondrial quality control (MQC). On the other hand, various forms of metabolism have emerged as important processes implicated in the regulation of MQC, including but not limited to processes such as mitochondrial biogenesis, mitochondrial dynamics (fusion and fission), and mitophagy. The multifaceted regulation of mitochondrial function by metabolites underscores a sophisticated cellular strategy to adapt to energetic demands and environmental stress, ensuring cell survival and optimal function. In this context, several recent studies have uncovered novel insights into how specific metabolites trigger distinct mechanisms via the formation of mitochondria-derived vesicles (MDVs) [1–3].

MDVs were first discovered in 2008, and normally have a diameter of 70−150 nm and contain various mitochondrial components, including mitochondrial DNA (mtDNA), lipids, and mitochondrial proteins [4]. They have been classified as a group of small vesicles protruding from mitochondria. In addition to their role in MQC, MDVs have also been associated with various physiopathological functions, spanning from antimicrobial defense to antigen presentation [5]. They could be produced under baseline conditions and generated rapidly in response to mitochondrial stress [5]. Two separate but complementary studies published earlier reveal that metabolites are also involved in the regulation of MDV biogenesis. Specifically, these studies demonstrate that pharmacological inhibition or genetic ablation of the tricarboxylic acid (TCA) cycle enzyme fumarate hydratase (FH) increases intracellular level of fumarate, which induces the biogenesis of MDVs dependent on sorting nexin 9 (SNX9), possibly via succination of mitochondrial proteins [2, 3]. These findings underscore the importance of metabolite fluctuations in the regulation of MQC via MDV formation. It remains elusive whether other mechanisms, particularly other types of metabolite post-translational modification (PTM), contribute to the biogenesis of MDVs.

**Figure 1:**
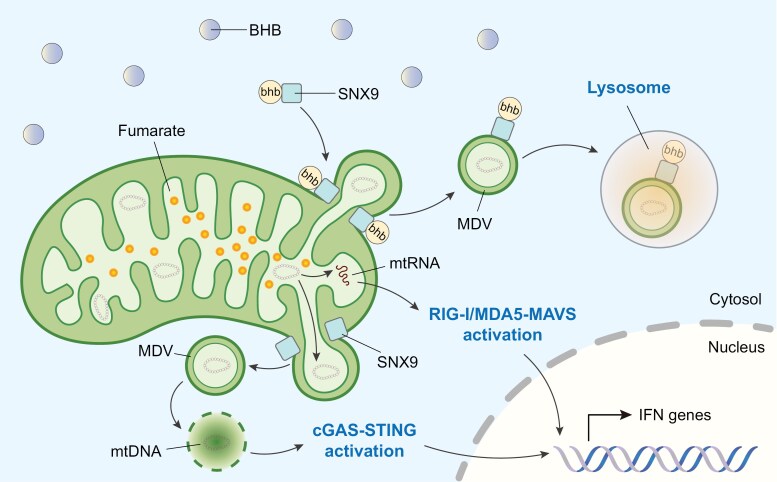
Metabolites influence SNX9-mediated MDV biogenesis. The enzyme FH plays a critical role in the conversion of fumarate to malate as part of the TCA cycle. However, in pathological conditions such as cancers and immune disorders, FH dysfunction leads to fumarate accumulation. This accumulation triggers SNX9-dependent MDV formation and the subsequent release of mitochondrial nucleic acids, including mtRNA and mtDNA, into the cytosol. The released mtRNA and mtDNA then activate the RIG-I/MDA5-MAVS and cGAS-STING pathways, respectively, resulting in the transcription of genes encoding type-I interferon proteins and subsequent inflammatory responses. Recently, Tang *et al*. discovered a novel role of BHB in the regulation of mitochondrial homeostasis. BHB promotes lysine Kbhb modification of SNX9, which facilitates the generation of MDVs. These MDVs are ultimately degraded in lysosomes, thereby restricting mtDNA-induced inflammation

In a recent study, through unbiased proteomic approaches, Tang *et al*. reported a novel PTM, namely lysine β-hydroxybutyrylation (Kbhb), on SNX9, which is critical for stress-induced MDV biogenesis [1] (Fig. 1). They discovered that β-hydroxybutyrate (BHB), the principal circulating ketone body, preserved mitochondrial functions via promoting the formation of MDVs in various hepatocellular carcinoma cell lines under mitochondrial damaging conditions. Mechanistically, BHB promoted Kbhb of SNX9, resulting in enhanced interaction of SNX9 with inner mitochondrial membrane (IMM)/matrix proteins and hence the formation of IMM/matrix MDVs. Subsequent interactome analysis revealed that two key IMM proteins, including optic atrophy 1 (OPA1) and stomatin-like protein 2 (STOML2), are bound to Kbhb-modified SNX9 to drive the biogenesis of MDVs. Furthermore, by mass spectrometry analysis, they identified three lysine residues, K202, K207, and K267, as sites of BHB-induced Kbhb modification on SNX9, while mutations of these sites to arginine (SNX9 3KR) impaired the critical functions of SNX9 in MDV biogenesis and mitochondrial function improvement. Functionally, they showed that BHB attenuated alcohol-induced liver injury in mouse models in a manner dependent on SNX9 Kbhb. Collectively, this study revealed Kbhb on SNX9 as a novel mechanism in regulating MDV generation and mitochondrial homeostasis.

The implications of the findings presented by Tang *et al*. are multifaceted. First, this study addressed a crucial scientific question: how do mitochondrial dynamics and metabolites reciprocally influence one another? BHB is the most abundant ketone body in human circulation and is produced by liver mitochondria. It is produced under metabolic stress such as prolonged fasting and ketogenic diets, or under pathological conditions like uncontrolled diabetes. As the key player in bioenergetics, mitochondria are highly sensitive to alteration of intracellular and extracellular metabolite levels, such as ATP, amino acids, and nicotinamide adenine dinucleotide (NAD^+^). Changes in these metabolites play a key role in modulating the mitochondrial functions. For instance, NAD^+^ finetunes mitochondrial homeostasis by initiating mitophagy for the clearance of damaged mitochondria [6]. Thus, the current study by Tang *et al*. provides compelling evidence that BHB directs the biogenesis of MDVs, through which the intracellular metabolites are bridged with the mitochondrial stress response. At present, whether and how metabolites such as BHB can regulate other forms of MQC, such as mitochondrial fusion and fission or mitochondrial biogenesis, is an important scientific question for future investigation.

Second, this study has uncovered a novel mechanism of how metabolites regulate the biogenesis of MDVs. While metabolites emerge as crucial players in promoting MDV biogenesis, the exact mechanisms remain elusive. Two recent studies reported that fumarate induces rapid succination of mitochondrial proteins [2, 3]. However, how this process impacts the generation of MDVs remains unclear. Thus, the work by Tang *et al*. places Kbhb modification of SNX9 at the center stage. They revealed that SNX9 Kbhb is indispensable for its binding to OPA1 and STOML2, thus enhancing the budding of MDVs from mitochondria. Of note, OPA1 is required for fusion of the IMM and has been shown to regulate IMM/matix MDV generation [7, 8]. This study thus provides mechanistic insights linking Kbhb modification of SNX9 to the biogenesis of MDVs, which represents a significant conceptual advance in that a metabolite can directly alter organellar function via metabolite PTM of proteins. It also broadens our understanding of how metabolic cues are intricately woven into the cellular machinery that preserves mitochondrial health.

Third, this study highlights that distinct metabolic conditions might preferentially activate different quality control pathways. Fumarate accumulation precipitates a pathological state marked by mitochondrial RNA (mtRNA) and mtDNA release and subsequent immune activation due to activation of the cyclic GMP-AMP synthase (cGAS )-stimulator of interferon genes (STING) pathway induced by mtDNA and the retinoic acid-inducible gene I (RIG-I)/melanoma differentiation-associated gene 5 (MDA5)-mitochondrial antiviral signaling protein (MAVS) pathway induced by mtRNA [2, 3] (Fig. 1). In contrast, the work by Tang *et al*. reveals that BHB serves as a protective agent that reinforces MQC. Instead of merely acting as a metabolic substrate, BHB engages a unique form of PTM that finetunes the function of key protein regulators like SNX9, thereby facilitating the selective removal of damaged mitochondrial components through the MDVs-lysosome pathway.

In summary, the study by Tang *et al*. reveals that MDVs bridge metabolite signals to mitochondrial fitness and such a notion naturally invites further research in multiple fronts. First, it remains to be further investigated how Kbhb modification of SNX9 directs MDVs for lysosomal degradation rather than releasing mitochondrial contents, such as mtDNA, into the cytosol. Second, it remains to be explored whether important metabolites such as BHB would affect other MQC pathways such as mitophagy, autophagic secretion of mitochondria, and mitolysosome exocytosis [9, 10]. Third, as the functions of mitochondria and other organelles are intricately interlinked, it will be of interest to explore the role of BHB and Kbhb modification in the quality control of other organelles, such as the endoplasmic reticulum. Last but not least, understanding this unique function of BHB and Kbhb in the biogenesis of MDVs and regulation of mitochondrial homeostasis opens new therapeutic avenues for a range of human diseases known to be associated with mitochondrial dysfunction.
